# Molecular docking and mouse modeling suggest CMKLR1 and INSR as targets for improving PCOS phenotypes by minocycline

**DOI:** 10.17179/excli2021-4534

**Published:** 2022-02-16

**Authors:** Mahdie Kian, Elham Hosseini, Tooba Abdizadeh, Taimour Langaee, Azadeh Khajouei, Sorayya Ghasemi

**Affiliations:** 1 Cellular and Molecular Research Center, Basic Health Sciences Institute, Shahrekord University of Medical Sciences, Shahrekord, Iran; 2 Department of Obstetrics and Gynecology, IVF Clinic, Mousavi Hospital, School of Medicine, Zanjan University of Medical Sciences, Zanjan, Iran; 3 Clinical Biochemistry Research Center, Basic Health Sciences Institute, Shahrekord University of Medical Sciences, Shahrekord, Iran; 4 Center for Pharmacogenomics and Precision Medicine, College of Pharmacy, University of Florida, Gainesville, FL, USA

**Keywords:** PCOS, INSR, CMKLR1, minocycline, inflammation

## Abstract

Polycystic ovary syndrome (PCOS) is the most common cause of women's infertility. Some inflammatory pathways play a pivotal role in the pathogenesis of PCOS. This study aimed to investigate the possible beneficial effects of minocycline on chemokine-like receptor 1 (CMKLR1) and Insulin Receptor (INSR) in a PCOS model. A molecular docking study was implemented using Molecular Operating Environment (MOE) software. The PCOS was induced in NMRI mice (mean body weight 14.47±0.23) by 28 days estradiol valerate injection (2 mg/kg/day). The mice were then divided into six groups (n=8 per group, mean body weight 17.77± 0.26): control (received normal saline), PCOS model, control for minocycline, minocycline treated PCOS (50 mg/kg), letrozole treated PCOS (0.5 mg/kg), and metformin-treated PCOS (300 mg/kg). Serum FSH, LH, estradiol (E2), and testosterone were detected by ELISA. The ovarian tissues were stained by hematoxylin and eosin. The CMKLR1 and INSR expression levels were determined by Real-time-PCR. The molecular docking studies showed scores of -10.92 and -9.30 kcal/mol, respectively, for minocycline with CMKLR1 and INSR. Estradiol valerate treatment led to a significant increase in E2, graffian follicle, and decrease in corpus luteum (CL) numbers (P<0.05), while minocycline treatment improved these PCOS features. The minocycline treatment significantly decreased the CMKLR1 expression and increased the INSR expression (P<0.05) while the CMKLR1 expression was increased in PCOS model. Minocycline may improve ovulation in PCOS model by returning E2 to a normal level and increasing CL number (ovulation signs). These beneficial outcomes may be related to the changes in CMKLR1 and INSR gene expression involved in glucose metabolism and inflammation.

## Introduction

Polycystic ovary syndrome (PCOS) is a genetic complex endocrine and metabolic disorder affecting 5 %-15 % of women of reproductive age (van Houten and Visser, 2014[48]). The main symptoms of PCOS are hyperandrogenism, ovulation disorders, and polycystic ovary morphology (PCOM) (Stein, 1935[43]). Obesity, an increased risk of developing heart disease, type 2 diabetes, and insulin resistance are common phenotypes in PCOS patients (Agarwal et al., 2020[2]; Glueck et al., 2004[17]). The insulin resistance in PCOS patients is exacerbated by obesity, resulting in impaired glucose tolerance that makes them susceptible to diabetes, metabolic syndrome, and insulin resistance (Diamanti-Kandarakis et al., 2006[10]). Furthermore, hormonal imbalances in luteinizing hormones (LH), androgens, and cortisol promote a low-grade chronic inflammation in these patients (Repaci et al., 2011[37]; Schmidt et al., 2014[39]). Several studies reported that PCOS patients present impaired levels of inflammatory markers (Lansdown and Rees, 2012[29]). In contrast, the reduced anti-inflammatory cytokines and antioxidant molecules account for chronic inflammatory responses (Estienne et al., 2019[12]; Zangeneh et al., 2017[56]), which affect in the long term female fertility and even risk of endometrial and breast cancer. 

Alteration in some endocrine compounds called adipokines is involved in the pathophysiology of the polycystic ovary. Among the various members of adipokines, the chemerin and its receptor, chemokine-like receptor 1 (CMKLR1), play an important role in the increased severity of PCOS (Bongrani et al., 2019[8]). The well-known dual role of chemerin protein in inflammation and metabolism of glucose is a possible underlying cause for insulin resistance in PCOS patients, mainly obese ones (Wang et al., 2019[51]; Yen et al., 2021[55]). Interestingly, the concentration and expression of chemerin in follicular fluid and granulosa cells are higher in PCOS cases than in normal women. CMKLR1 probably mediates meiotic progression blockade at the germinal vesicle stage of oocyte and decreases steroidogenesis synthesis of ovarian cells. So, deregulation expression of chemerin may play a role in ovulatory dysfunction and the arrest of follicular growth (Lin et al., 2021[33]). 

Additionally, chemerin induces insulin resistance by disrupting the insulin receptor (INR) signaling pathway and glucose uptake. In this context, insulin-sensitizing drugs like metformin (the first-line of treatment for PCOS patients with insulin resistance) are able to decrease chemerin serum levels and inhibit the harmful effects of hyperinsulinemia (Yen et al., 2021[55]). As mentioned, a high serum level of chemerin is related to several different disorders. Therefore, it seems that there is a causative association between chemerin and its corresponding receptor, CMKLR1, and several manifestations of PCOS (Serafin et al., 2019[40]). It has been shown that the PCOS treatment by conventional and/or new therapeutic approaches can improve the metabolic parameters. These treatments are based on the patient's needs; some of them target the hyperandrogenism's manifestations, others are restricted to alleviate insulin-resistant symptoms, and some treatment methods focus on the underlying chronic inflammatory processes (Bastos et al., 2007[5]; Grotegut et al., 2020[20]). So, it seems that minocycline may be a better choice for treatment with multiple functions to reduce comorbidities in PCOS patients (Goldsmith and Dowd, 1993[18]).

Hyperandrogenemia, the most important complication of PCOS, is one of the leading causes of acne and menstrual problems in adolescent women. The positive effects of minocycline on the elimination of bacterial-caused acne and skin lesions and some anti-inflammatory properties make this drug a suitable medication for the management of some phenotypes in PCOS patients (Moghetti and Toscano, 2006[35]). It seems that there is a strong association between the chemerin and insulin resistance signaling pathways that are involved in both inflammation and metabolic defects in PCOS pathology (Serafin et al., 2019[40]; Xie et al., 2020[53]). In order to better manage the PCOS syndrome, the study of the first receptors of these two pathways and the selection of an approved drug without side effects are helpful.

In this study, we first applied molecular docking screening using Molecular Operating Environment (MOE) software to study the interactions between minocycline and CMKLR1 and INSR modeled proteins. Then, we investigated the potential therapeutic effects of minocycline on the expression of two effective genes (CMKLR1 and INSR) in inflammation and glucose metabolism, hormonal profile, and ovarian histology in the PCOS NMRI mouse model.

## Materials and Methods

### Homology modeling and the target-template sequence alignment

The experimental crystal structures of mouse CMKLR1 and INSR are not available in the Protein Data Bank (PDB) yet; therefore, the homology modeling approach was used to obtain 3D models (Biasini et al., 2014[7]). The FASTA sequences of CMKLR1 and INSR (accession numbers: P97468 and P15208, respectively) were retrieved from the Uniprot knowledge base (http://www.uniprot.org). These protein sequences were used to find suitable templates with the most similarity to these proteins by the PSI-BLAST (Kumar et al., 2018[28]), JPRED4 (Shehadi et al., 2020[41]), and Phyre2 against PDB database.

The selected templates for CMKLR1 and INSR were 6OMM and 6PXV, respectively (Fiser and Šali, 2003[14]), on the basis of maximum query coverage, maximum identity, high score, and lower e-value for homology modeling. The Clustal W was used for alignment of the amino acid sequences of homology models of CMKLR1 and INSR with the template structures for the homology model (Altschul et al., 1990[3]). Then homology modeling of CMKLR1 and INSR were carried out by the SWISS-MODEL server (http://swissmodel.exposy.org). 

### Structure validation of modeled proteins

The SWISS-MODEL server calculates the QMEAN scoring performance to estimate the local and global model quality based on the geometry, the interactions, and solvent the potential of the protein model (Benkert et al., 2008[6]; Glueck et al., 2004[17]). The quality of the generated models by SWISS-MODEL was evaluated by using various validation techniques such as Rampage server to analyze the Ramachandran plot (Colovos and Yeates, 1993[9]; Hart et al., 2004[21]) and ProSA, which determine the z-score of modeled structures to evaluate the protein quality of the models (Elting et al., 2003[11]). ERRAT (Apter, 1998[4]), the statistics of nonbonding interactions between different atom types are analyzed for a structure with a sliding window of nine residues, and the generated models are verified by GMQE scores, QMEAN values and 3D QMEAN (Wiederstein and Sippl, 2007[52]). Additionally, the structures of the constructed CMKLR1 and INSR models and templates were compared using Root Mean Squared Deviation (RMSD) by UCSF chimera 1.10.

### Prediction of active sites in the modeled proteins

The active sites of the modeled proteins were predicted using the Computed Atlas of Surface Topography of Proteins 3.0 (CASTp) (Laven et al., 2004[30]). By sending modeled 3D proteins to the server, CASTp identified the active site of the proteins and also predicted the amino acids necessary for binding interaction by the submission of the modeled 3D proteins on the server. 

### Molecular docking study

Molecular docking analysis was carried out using Molecular Operating Environment (MOE) software to study the interactions between minocycline and CMKLR1 and INSR modeled proteins. The 2D structure of minocycline was built using ChemDraw Ultra 12.0 software, and its 3D structure was obtained by Hyper Chem 7 software (Hyper cube Inc., USA) using molecular machine force pre-optimization field and followed by semi-experimental calculation of AM1. Best models obtained from SWISS-MODEL in PDB format were used for molecular docking studies (Lengauer and Rarey, 1996[31]). The structure of modeled proteins was subjected to 3D protonation and energy minimization using the following parameters, including force field of MMFF94X and solvation, chiral constrain of current geometry and gradient of 0.05. Docking was performed with default parameters of MOE and free energies of minocycline binding with the CMKLR1 and INSR proteins and obtained by MOE docking score. For minocycline, the top-score docking poses were selected for final ligand-target interaction analysis by the LigX module in MOE (Kitchen et al., 2004[26]). 

### Model samples preparation

A total of 48 NMRI mice (average body weight 14.47 ± 0.23) were purchased from the Pasteur Institute (Tehran, Iran). All procedures and animal care were performed according to the Guide for the Care and Use of Laboratory Animals (NRC, 2011[36]). The study was approved by the ethic committee of Shahrekord University of Medical Sciences (SKUMS. REC.1398.008). The mice were kept in a temperature-controlled laboratory environment (12-hour light/dark cycle, 25 °C) and given free access to chow and water. The PCOS modeling was created in the previous study and related parameters for confirming the PCOS model were reported elsewhere; however, the procedures are described briefly here and in detail by Khajouei et al. (2021[25]) except that an extra group (metformin group) was also created in the current study. 

A summary of the report from our previous study for modeling and its confirmation is reviewed here. Mice were weighted again 28 days after the estradiol valerate (EV) injection for PCOS induction, then randomly divided into six groups (n = 8 per group), including the control group (IP injection of normal saline for 28 days), PCOS model group (IP injection of 2 mg/kg EV daily for 28 days), Minocycline control group (IP injection of normal saline for 28 days continued by 50 mg/kg minocycline injection for 10 days), the minocycline model group (IP injection 2 mg/kg EV for 28 days for PCOS modeling continued by 50 mg/kg minocycline for 10 days). The positive control group for CMKLR1 gene was letrozole model (28 days IP injection of 2 mg/kg EV, then S.C injection of 0.5 mg/kg letrozole for another 28 days). The positive control group for the INSR was the metformin model (IP injection of 2 mg/kg EV, for 28 days, then gavage of 300 mg/kg metformin for 14 days). Vaginal smear samples were taken at 9:00 AM every day from mice and stained with Giemsa (Merck, Germany #109204). The slides were evaluated under a light microscope, and different phases of the estrous cycle were diagnosed based on the dominant cells present in the samples. At the end of the interventions, animals were euthanized, weighted, and blood was collected transcardially, and serum was collected by centrifugation (at 3000 g for 10 minutes) and stored at -20 °C for further analysis. ELISA kits were used to assess testosterone, FSH, LH, and Estradiol (E2) levels (Monobind Inc. method) (Khajouei et al., 2021[25]). The weight of mice was followed-up weekly during the experiment procedures. However, body weight was reported twice: after PCOS induction, mice with comparable body weight were randomly divided over six treatment groups for confirmation of the same allocation of them in the different groups, and at the end of treatment to ensure that the mice were all assigned to the same groups. 

### Evaluation of tissue histology and morphology

Following the collection of blood samples, ovaries were removed and fixed in 4 % paraformaldehyde buffer. After routine histological procedures, the ovaries were embedded in paraffin, cut into 5 μm sections, mounted on the slid and stained by hematoxylin and eosin (H&E) dye (Merck, Germany, #104302 and #115935, respectively). A digital camera mounted on a light microscope (Eclipse E200 microscope) and imaging software. NIS-Elements imaging software AR was used to capture images from the slides. From three randomly selected slides of each ovary, the different types of follicles (primary, secondary, antral, and atretic follicles) and corpus luteum (CL) were counted. 

### Quantitative real-time-PCR 

One frozen ovary from each mouse was placed in TRIzol® (GeneAll, #301-001) reagent. The remaining processes were carried out in accordance with the manufacturer's instructions. The quantity and quality of RNA were measured using the NanoDrop 2000 spectrophotometer (Thermo Fisher Scientific, Carlsbad, CA, USA). The cDNA was synthesized from 1 µg of total RNA, random hexamers, using a commercial cDNA synthesis kit (Perma Tajhiz Azma, Iran). The mRNA expression was quantitated on the Rotor-Gene 3000 device using primers and RealQ Plus 2x Master Mix Green (Amplicon, Denmark), according to the manufacturer's protocol.

The RPLP0 endogenous gene was used as internal control, and gene expression levels were calculated using the 2-^ΔΔCt^ method (Fu et al., 2010[15]). The primers were designed using the PRIMER3.0 program (http://bioinfo.ut.ee/primer3-0.4.0). The primer specificity was verified by using Primer blast tool from the NCBI genome browser. Table 1(Tab. 1) shows the primer sequences used for Real-time PCR assays. 

### Analytical statistics

The findings were analyzed with SPSS 22.0 and GraphPad Prism version 2.0.8 statistical software and presented as mean ± SEM. The impact of treatments in different groups was investigated using one-way analysis of variance (ANOVA) by Tukey's post hoc test. Statistical significance was described as a P value of less than 0.05. Each test was carried out at least three times.

## Results

### Homology modeling

The homology modeling is a reliable and acceptable technique for predicting and generating protein structure using a template. The amino acid sequences of CMKLR1 (P97468) and INSR (P15208) were obtained from the Uniprot database and submitted to PDB-BLAST, Jpred4, and Phyre2 server tools for identifying appropriate templates for homology modeling. The crystal structure of N-formyl peptide receptor 2 (PDB ID: 6OMM) was selected as the most suitable template for CMKLR1 with sequence identity of 35.19 %, query covering of 89 %, Max score 201 and statistical E-value of 6e-61. The insulin receptor (PDB ID: 6PXV) was identified as the best template for INSR with a sequence identity of 95.64 %, query coverage of 98 %, Max score of 2670 and statistical E-value of 0.0 (Supplementary Table 1). The 3D structures of CMKLR1 and INSR were created using SWISS-MODEL with GMQE of 0.56 and 0.66 and QMEAN of -5.16 and -2.03 respectively (Vutyavanich et al., 2007[50]). These scores showed that the modeled structures were reliable with good quality. The results of the amino acid sequence alignment of CMKLR1 and INSR with the templates 6OMM and 6PXV by Clustal W are shown in Supplementary Figures 1 and 2 and these alignments indicated that the targets and template proteins were closely related in origin. According to these results, the sequence alignments of the CMKLR1 and 6OMM showed 35.19 % sequence identity and INSR and 6PXV showed 95.46 % sequence identity. The RMSD values of 0.181 and 0.224 Å obtained and computed from the alignment using Chimera showed that the templates and generated models had high structural similarity with low RMSD values (Supplementary Figures 3, 4). 

### Validation of modeled protein structures

The Ramachandran plot was obtained from Rampage for modeled CMKLR1 showed that 92.9 % of residues were in most favored regions, 6.0 % of residues in additional allowed regions, 0.4 % of the residues in generously allowed regions and 0.7 % of the residues in disallowed regions (Supplementary Figure 5). Ramachandran plot statistics for modeled INSR showed a total 85.0 %, 12.6 %, 1.5 %, and 0.9 % residues in most favored, additionally allowed, generously allowed and disallowed regions, respectively (Supplementary Figure 6). The plots of the predicted local similarity to targets against the residue number of the predicted 3D structures of the modeled proteins are graphically shown in (Supplementary Figure 7). The values of most of the residues were close to 1, indicating that the local quality estimate of the residues of the predicted models was good. The residues' protein structures also lie within the range of other protein structures in PDB, which confirms the reliability. These plots showed that no bad scores were for main-chain or side-chain parameters.

The Z-score from ProSA tool was used to evaluate the model's quality. The Z-scores for CMKLR1 and INSR models were -2.51 and -10.51, respectively, which are within the acceptable ranges according to the X-ray (light blue zone) and NMR structure (dark blue region). The most of CMKLR1 and INSR residues had negative ProSA energies and ERRAT scores of 99.63 and 85.73 for CMKLR1 and INSR showed that backbone conformation and non-bonded interactions of the models were acceptable (Supplementary Figures 8, 9).

The results from the validation methods showed that, the homology models of CMKLR1 and INSR were satisfactory and reliable for further studies. The results of molecular docking analysis were confirming the level of binding site of the modeled proteins calculated using the CASTp in CMKLR1 and INSR (Supplementary Figures 10, 11). 

### Molecular docking studies

**Figure 1:**
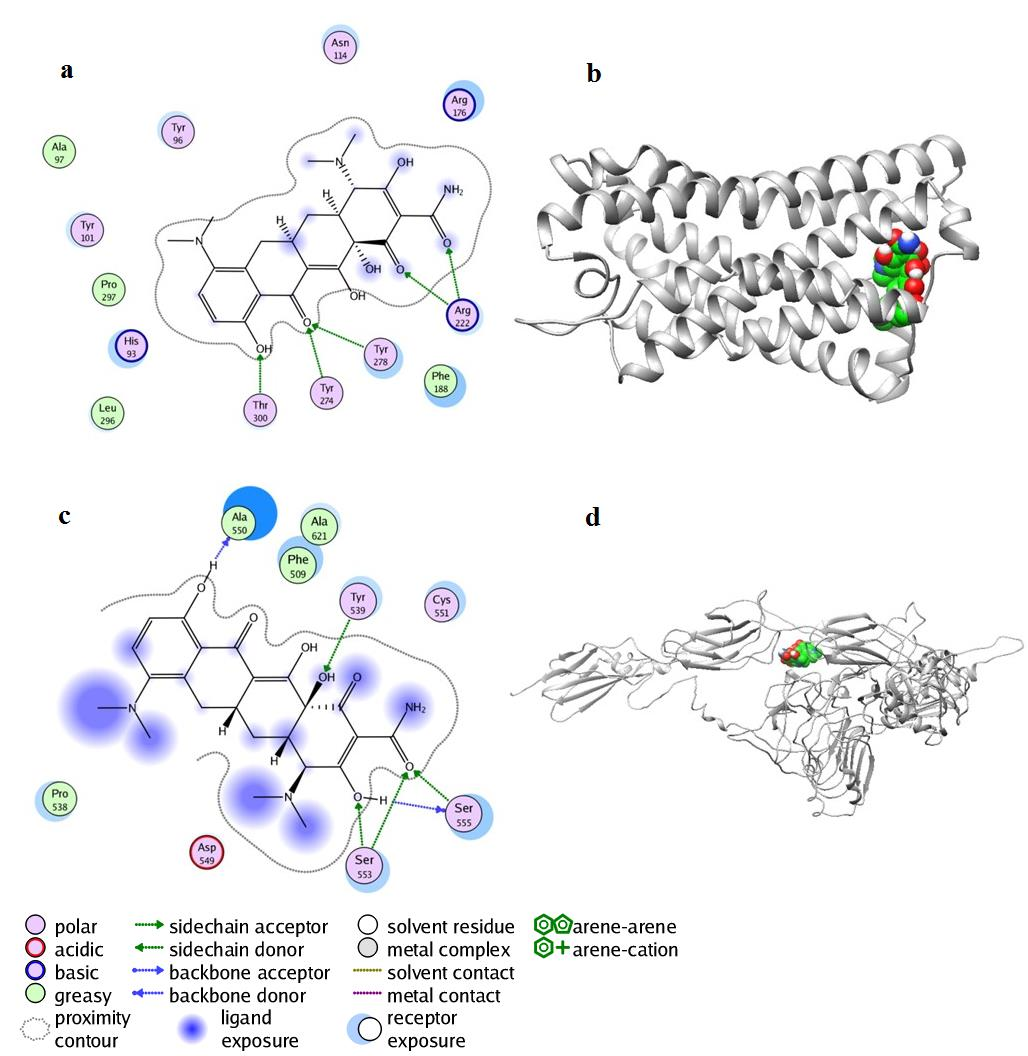
Molecular docking results: (a) two-dimensional display of interaction between CMKLR1 and minocycline. (b) Three-dimensional interaction between CMKLR1 and minocycline. (c) Two-dimensional display of the interaction between INSR and minocycline. (d) 3D displays of the interaction between INSR and minocycline

The docking results showed that docking scores of the minocycline and CMKLR1 and INSR were -10.92 and -9.30 kcal/mol respectively. The interaction of the minocycline in the active site of the modeled proteins showed that the minocycline was well established and had significant interactions with the key amino acids of these proteins. Investigation of the interaction of the minocycline with CMKLR1 by LigX module of MOE showed that carbonyl and hydroxyl groups of minocycline could form hydrogen bonds with amino acid residues; Arg222, Tyr278, Tyr274 and Thr300. The phenyl and cyclohexyl rings in minocycline had hydrophobic interactions with Phe188, Leu296, Ala97, and the electrostatic interactions with Tyr96, Tyr101, Arg176, His93 and Asn114 (Figure 1a, 1b(Fig. 1)). 

Within the active site of INSR with minocycline, carbonyl and hydroxyl groups of the minocycline could form six hydrogen bonds with Ala550, Tyr539, Ser553 amino acids. Also, phenyl and cyclohexyl rings of the minocycline in the active site of INSR had hydrophobic interactions with Phe550, Ala621, and Pro538 and the electrostatic interactions with amino acid residues, Cys551 and Asp549 (Figure 1c, 1d(Fig. 1)). The molecular docking studies presented the possible binding mode of the minocycline at the active site of CMKLR1 and INSR by Thr, Ser, Arg, and Tyr amino acids using hydrogen bonds. The results from the interaction of CMKLR1 and INSR proteins with the minocycline could be used to further study the molecular mechanisms.

### Evaluation of the estrous cycle and serum hormone profile for confirming mouse modeling

**Figure 2:**
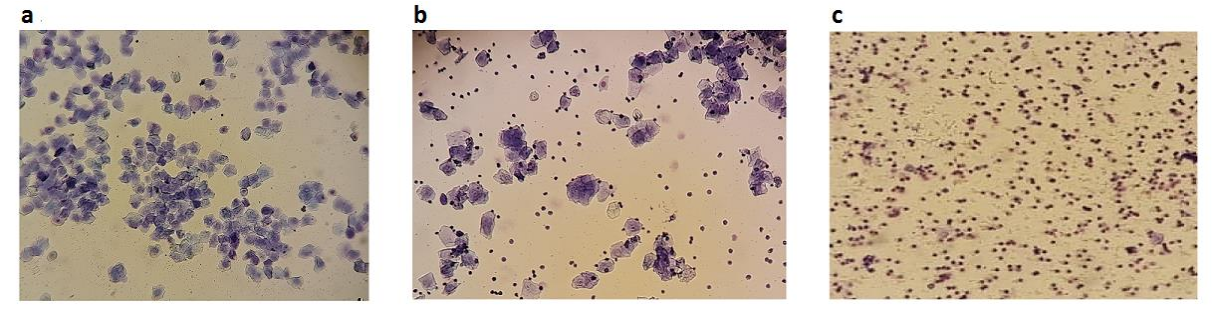
Determining the estrous cycle stages by vaginal smear/cytology: a) Estrus stage: non-nucleated surface cells. b) Metestrus stage: with non-nucleated surface cells, leukocytes, and epithelial cells. c) Diestrus stage: identified by predominant presence of leukocytes (Magnification, 4x)

The parameters used to confirm the mouse PCOS modeling was reported before (Khajouei et al., 2021[25]), and in this study metformin group was also confirmed. The estrous cycles of all mice in the control group who received normal saline for 28 days were in the four regular phases (proestrus, estrus, metestrus, and diestrus) (Figure 2(Fig. 2)), but the estrous cycles of the mice who received EV for 28 days (PCOS model) were irregular, spending more days in the estrus stage. After one cycle of treating the PCOS mice with minocycline, the analysis showed that the estrous cycle became regular to some extent.

Furthermore, as reported previously (Khajouei et al., 2021[25]), the levels of LH, FSH, and T did not change significantly between the groups. In comparison to the control group, E2 was significantly higher in the PCOS model group (P<0.01). Following treatment with minocycline, letrozole, and metformin, E2 levels decreased significantly in comparison with the PCOS model (P<0.01). Supplementary Table 2 shows the hormone profile of the metformin group compared to the other studied groups. 

The mean body weight of all mice was 17.77±0.26 before the beginning of PCOS induction, so there were no differences in body weight across the groups. At the end of the study, the mean body weight was 22.90± 0.24, which did not differ significantly, as well.

### Results of histological studies 

**Figure 3:**
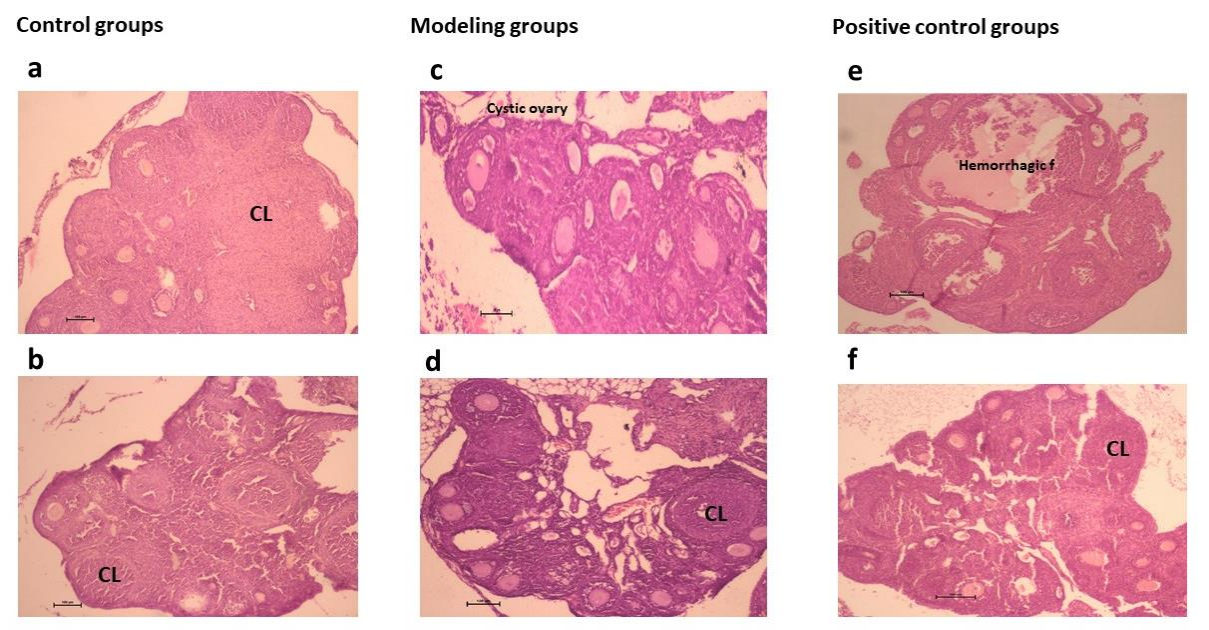
Histological sections of ovarian tissues of the treated groups in comparison with the control groups assessed by Hematoxylin and Eosin staining method (H&E): Grouping as follows: (a) control, (b) minocycline control, (c) PCOS model, (d) PCOS minocycline, (e) PCOS letrozole, (f) PCOS metformin. CL: Corpus luteum, GF: Graafian follicle (Magnification, 4X and 10X)

A large number of antral follicles were observed in the PCOS model (a sign of ovarian cysts) (Figure 3(Fig. 3)). The number of CL in the PCOS model group (as a sign of ovulation) showed a significant decrease compared to the control group (P<0.001). Furthermore, its number in the PCOS minocycline showed a significant increase in comparison with the PCOS model (P<0.01), which was similar to ovarian responses in the metformin group. The number of Graafian follicles was significantly increased in the PCOS model vs. control, and in contrast, were decreased in the PCOS models treated by minocycline, letrozole, and metformin. The data of ovarian follicle evaluation in different stages of studied groups were shown in Supplementary Table 3.

### Evaluation of CMKLR1 and INSR gene expression level in the studied groups 

**Figure 4:**
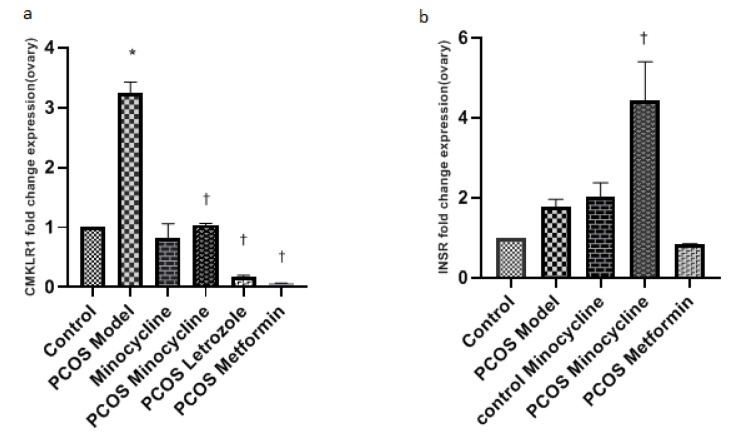
Comparison of CMKLR1 and INSR gene expression in different experimental groups. a) The expression of the CMKLR1 gene in the PCOS increased significantly compared to the control (*: P<0.0001) whereas, its expression in the PCOS treated by minocycline and letrozole showed a significant decrease compared to the PCOS model group (†:P<0.0001). b) INSR gene expression significantly increased in the PCOS treated with minocycline vs. PCOS model (†: P<0.001). The results were measured by One-Way ANOVA and presented mean ± SEM.

The CMKLR1 gene expression level significantly increased in the PCOS model compared to the control group (P< 0.0001). The CMKLR1 gene expression was reduced in the PCOS group treated with minocycline as compared to the PCOS model. Furthermore, in both letrozole and metformin PCOS treated groups the CMKLR1 gene expression was reduced as well (Figure 4a(Fig. 4)). 

The INSR gene expression in the PCOS model was similar to the control and the metformin group. In contrast, its expression was significantly increased in the PCOS treated with minocycline vs. PCOS model (Figure 4b(Fig. 4)).

See also the Supplementary data.

## Discussion

The cardinal presenting symptoms of PCOS include anovulatory cycles, elevated androgen levels, and ovaries with polycystic morphology, all of which are used as diagnostic criteria for this condition. Insulin resistance, obesity, and diabetes contribute to PCOS onset (Toosy et al., 2018[47]). Moreover, several studies have reported that patients with PCOS are at risk for low-grade chronic inflammation; however, the specific underlying molecular mechanisms and their consequences remain to be fully understood (Aboeldalyl et al., 2021[1]; Xiong et al., 2011[54]). 

In the present study, we focused on applying minocycline as a medication being used to treat acne in PCOS (Maffeis and Veraldi, 2010[34]). We selected two pleiotropic factors, chemerin and insulin, and performed a molecular docking study to determine any interactions between minocycline and CMKLR1 and INSR as the respective receptors of chemerin and insulin. Molecular docking was carried out on the minocycline compound with modeled CMKLR1 and INSR proteins using MOE software. Thus, functional and structural insights into the binding of minocycline and the modeled CMKLR1 and INSR proteins were obtained. The docking analysis provided a number of configurations that were scored to determine favorable binding modes. The docking study showed that minocycline has a putative binding mode at the active site of both receptors, so minocycline was well established and had significant interactions with the key amino acids of these proteins.

Mouse PCOS models were prepared by EV injection and confirmed by evaluating estrous cycles in the next step. The proportion of time spent in the estrous stage in the pre-treatment study significantly increased in EV-exposed mice compared to those not exposed, as previously reported (Khajouei et al., 2021[25]). Estrous cyclicity disruption, mostly arrested in the estrous phase, has been proven to create mouse PCOS models (Kauffman et al., 2015[24]). Little or no corpus luteum formation in the ovaries is another feature of such a model, and both were created and confirmed in our study (Supplementary Table 3). 

Chemerin, a member of the adipokines family, has pleiotropic effects on different cell types, and based on the type of tissue or physiological and biological activity, its functions are associated with specific receptors, mainly CMKLR1 (Helfer and Wu, 2018[22]). It has been recently shown that chemerin is involved in regulating female reproductive processes, acting as a regulator in linking metabolic function, steroidogenesis, and reproductive functions in the ovaries (Singh et al., 2018[42]). 

Both chemerin and its receptor are expressed in ovarian cells. Studies have shown that chemerin's follicular fluid concentration is higher than its plasma levels, suggesting that its ovarian and systemic regulations are different and indicating that it could play a role in local paracrine/autocrine regulation at the ovarian level (Singh et al., 2018[42]). Elevated serum levels of chemerin are associated with PCOS and some features of the metabolic syndrome, such as obesity and insulin resistance. Some of these features are related to anovulatory cycles in PCOS patients and others to insulin resistance (Bongrani et al., 2019[8]; Estienne et al., 2019[12]). Bovine granulosa cells cultured with chemerin showed a decrease in steroidogenesis and an arrest in antral follicle growth, resulting in a blockage in the progression of the oocyte meiotic process via the function of the chemerin receptor, CMKLR1 (Reverchon et al., 2014[38]). These alterations can lead to anovulatory cycles. On the other hand, a high level of this hormone in the serum can compensate for insulin resistance (Tan et al., 2009[44]). In the current study, the mRNA expression of CMKLR1 in ovarian tissues showed a significant increase in the PCOS model group compared to the control group (Figure 4a(Fig. 4)), which is in line with the studies above.

The present study investigated the effect of minocycline therapy to determine whether it can modulate manifestations of PCOS other than acne through its synergistic effects.

Minocycline has been shown to have both anti-inflammation and anti-diabetic properties. These properties make minocycline an alternative medicine for diabetes mellitus, where inflammation plays a key role (Garrido-Mesa et al, 2013[16]; Viana et al., 2014[49]). The current results showed that EV mouse treatment leads to a significant increase in E2 levels and Graafian follicle numbers and a decrease in CL. In contrast, minocycline treatment increased CL numbers, which shows restored ovulation after treatment (Supplementary Table 3). 

The chemerin serum level increases in PCOS patients, especially in those who are obese. After metformin therapy for three months (as the first-line of treatment for insulin resistance), chemerin and insulin serum concentrations significantly decrease in such cases (Yen et al., 2021[55]). In the current study, after minocycline therapy in the PCOS model, CMKLR1 expression significantly decreased compared to the PCOS model, as its expression was compatible with the control group (Figure 4a(Fig. 4)). Interestingly it has been shown that the PCOS progression can be prevented by CMKLR1 targeting or inhibition (Tang et al., 2016[45]). Physiological levels of chemerin play a pivotal role in promoting follicle growth and preserving the immunological homeostasis of the ovarian tissues. However, its increased production under hyperandrogenic conditions (in PCOS cases) induces the movement of some monocytes expressing CMKLR1 from the circulation system towards the ovary. During these processes, mononuclear cells are attracted to chemerin-rich ovarian tissues and differentiate into macrophages; subsequently, cell apoptosis occurs, especially in granulosa cells (Lima et al., 2018[32]). Minocycline can also inhibit immune activation by reducing monocytes and the secretion of pro-inflammatory cytokines (Kobayashi et al., 2013[27]). These changes lead to the suppression of diseases. Therefore, changing the pro-inflammatory environment into an anti-inflammatory one using minocycline leads to the CMKLR1+monocyte reduction in PCOS patients. Following that, the influence of elevated levels of chemerin is neutralized (Lima et al., 2018[32]). This study confirmed that, firstly, minocycline could interact with CMKLR1 (molecular docking study); secondly, it may reduce its gene expression, by which the development of PCOS feature is prevented. 

On the other hand, it has been shown that INSR polymorphism or changes in its gene expression possibly play an essential role in PCOS predisposition. This happens in insulin resistance-related pathways, and this gene may be considered a PCOS risk gene (Tian et al., 2020[46]). Moreover, insulin sensitivity is decreased in PCOS granulosa cells, impairing the glucose uptake process. The increase in chemerin level aggravates insulin resistance in such patients and leads to aberrations in ovarian steroidogenesis accompanied by anovulation. Altogether, these findings suggest a new role for chemerin in treating metabolic disorders, especially in PCOS (González, 2012[19]). In addition, the ovarian chemerin gene expression in animal PCOS models is attenuated after metformin treatment. This treatment finally resulted in the improvement of insulin resistance and steroid levels (Kabiri et al., 2014[23]). Based on our results, INSR expression increased significantly after minocycline treatment in the PCOS mouse model. Since the INSR gene expression was significantly increased in the minocycline-receiving model group, it seems that minocycline and estradiol valerate synergistically affect the INSR gene. It is possible that the two most important factors involved in the disease, chemerin and insulin resistance, are ameliorated by this drug, leading to the improvement of the disease symptoms. In this context, as chemerin directly relates to provoking insulin resistance and adiposity, other studies suggest that it can be considered as an interesting clinical indicator for metabolic disorders (Fatima et al., 2013[13]; Singh et al., 2018[42]). It is recommended to study the expression of target genes at the protein level. 

## Conclusion

Minocycline treatment of the PCOS animal model showed that it might improve some symptoms of the syndrome by affecting the expression level of chemerin and insulin receptors and returning E2 to a normal level. Since the standard management of such patients seems to be inefficient, the synergistic action of minocycline on ovulation induction and traditional acne treatment may be an excellent alternative medication in such patients as it may reduce the need for additional drugs in patients; our findings warrant further studies.

## Notes

Mahdie Kian and Elham Hosseini contributed equally as first author.

## Declaration

### Acknowledgments

The authors thank the staff of the Cellular and Molecular Research Center and research deputy of Shahrekord University of Medical Sciences. We also thank Dr. Gholamreza Mobini and Dr. Hossein Amini for their help on this study.

### Funding source

This project was supported by a research grant (Grant No. 3011) from Shahrekord University of Medical Sciences IR.SKUMS.REC.1398.008.

### Authors' contribution

SG conceived the study, edited the paper, coordinated, and received a grant. MK performed experiments and wrote the paper draft. AK performed experiments. EH wrote the paper and analyzed data. TA performed the docking study. TL wrote and edited the paper. 

### Conflict of interest

The authors declare that they have no conflict of interest.

## Supplementary Material

Supplementary information

Supplementary data

## Figures and Tables

**Table 1:**
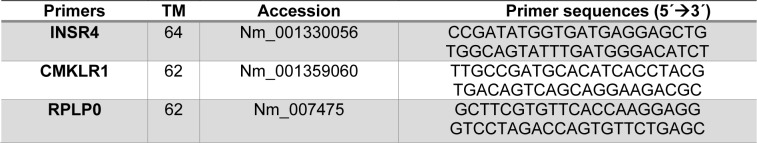
Primer sequences used for real-time PCR
