# Supplementary material for: Molecular docking and mouse modeling suggest CMKLR1 and INSR as targets for improving PCOS phenotypes by minocycline
Source: EXCLI J. 2022 Feb 16;21:400–14. doi: 10.17179/excli2021-4534 (PMC8971357; doi:10.17179/excli2021-4534)
Supplement: Supplementary information [file EXCLI-21-400-s-001.pdf]

EXCLI Journal 2022;21:400-414 – ISSN 1611-2156
Received: November 20, 2021, accepted: February 09, 2022, published: February 16, 2022

Original article:

MOLECULAR DOCKING AND MOUSE MODELING SUGGEST CMKLR1 AND INSR AS TARGETS FOR IMPROVING PCOS PHENOTYPES BY MINOCYCLINE

Mahdie Kian[a†], Elham Hosseini[b†], Tooba Abdizadeh[c], Taimour Langaee[d], Azadeh Khajouei[a], Sorayya Ghasemi[a*]

[a] Cellular and Molecular Research Center, Basic Health Sciences Institute, Shahrekord University of Medical Sciences, Shahrekord, Iran
[b] Department of Obstetrics and Gynecology, IVF Clinic, Mousavi Hospital, School of Medicine, Zanjan University of Medical Sciences, Zanjan, Iran
[c] Clinical Biochemistry Research Center, Basic Health Sciences Institute, Shahrekord University of Medical Sciences, Shahrekord, Iran
[d] Center for Pharmacogenomics and Precision Medicine, College of Pharmacy, University of Florida, Gainesville, FL, USA

† Joint first authors, contributed equally to this research work.

* **Corresponding author:** Sorayya Ghasemi, Ph.D., Associate Professor, Cellular and Molecular Research Center, Basic Health Sciences Institute, Shahrekord University of Medical Sciences, Shahrekord, Iran; Tel: 09131856090, 03833331471;
E-mails: s.ghasemi@skums.ac.ir, sorayya.ghasemi@gmail.com

https://dx.doi.org/10.17179/excli2021-4534

This is an Open Access article distributed under the terms of the Creative Commons Attribution License (http://creativecommons.org/licenses/by/4.0/).

Received: November 20, 2021, accepted: February 09, 2022, published: February 16, 2022

```
sp|P97468|CML1_MOUSE      MEYDAYND-SGIYDDEYSDGFGY------FVDLEEASPWEA---KVAPVFLVVIYSLVCF      50
pdb|6OMM|R                -DYKDDDDVDGSAENLYFQGASMETNFSTPLNEYEEVSYESAGYTVLRILPLVVLGVTFV      59
                           :*.  :* .*  :: * :* .        ::  *   :*:   .*  :: :*: .:. .

sp|P97468|CML1_MOUSE      LGLLGNGLVIVIATFKMKKTVNTVWFVNLAVADFLFNIFLPMHITYAAMDYHWVFGKAMC      110
pdb|6OMM|R                LGVLGNGLVIWVAGFRMTRTVTTICYLNLALADFSFTATLPFLIVSMAMGEKWPFGWFLC      119
                          **:******* :* *:*.:**.*: ::***:*** *.  **: *.  **. :* **  :*

sp|P97468|CML1_MOUSE      KISNFLLSHNMYTSVFLLTVISFDRCISVLLPVWSQNHRSIRLAYMTCSAVWVLAFFLSS      170
pdb|6OMM|R                KLIHIVVDINLFGSVFLIGFIALDRCICVLHPVWAQNHRTVSLAMKVIVGPWILALVLTL      179
                          *: ::::. *:: ****: .*::****.** ***:****:: **  .  . *:**:.*:

sp|P97468|CML1_MOUSE      PSLVFRDTANIH-GKITCFNNFSLAAPESSPHPAHSQVVSTGYSRHVAVTVTRFLCGFLI      229
pdb|6OMM|R                PVFLFLTTVTIPNGDTYCTFNFAS--WGGTPEER----LKVAITMLTARGIIRFVIGFSL      233
                          * ::*  *..*  *.  *  **:     .:*.      :... :  .*  : **: ** :

sp|P97468|CML1_MOUSE      PVFIITACYLTIVFKLQRNRLAKNKKPFKIIITIIIITFFLCWCPYHTLYLLELHHTAV--      287
pdb|6OMM|R                PMSIVAICYGLIAAKIHKKGMIKSSRPLRVLTAVVASFFICWFPFQLVALLGTVWLKEML      293
                          *: *:: **  *. *:::: : *..:*:::: ::: :**:** *:: : **

sp|P97468|CML1_MOUSE      ---PSSVFSLGLPLATAVAIANSCMNPILYVFMGHDFRKFKVAL-FSRLANALSEDTGPS      343
pdb|6OMM|R                FYGKYKIIDILVNPTSSLAFFNSCLNPMLYVFVGQDFRERLIHSLPTSLERALSEDSAPT      353
                               .::.: :  ::::*: ***:**:****:*:***:  :    : * .*****:.*:

sp|P97468|CML1_MOUSE      SYPSHRSFTKMSSLNEKASVNEKETSTL      371
pdb|6OMM|R                NDTAANSASP------------------      363
```

Supplementary Figure 1: Alignment of CMKLR1 amino acid sequence and 6OMM crystalline structure. * Shows fully protected root positions (:) It represents protection between groups with quite similar characteristics, (.) indicating protection between groups with low similarity characteristics.

EXCLI Journal 2022;21:400-414 – ISSN 1611-2156
Received: November 20, 2021, accepted: February 09, 2022, published: February 16, 2022

```
sp|P15208|INSR_MOUSE    MGFGRGCETTAVPLLVAVAALLVGTAGHLYPGEVCPGMDIRNNLTRLHELENCSVIEGHL    60
pdb|6PXV|A              ---------------------------HLYPGEVCPGMDIRNNLTRLHELENCSVIEGHL    33
                                                   *********************************

sp|P15208|INSR_MOUSE    QILLMFKTRPEDFRDLSFPKLIMITDYLLLFRVYGLESLKDLFPNLTVIRGSRLFFNYAL    120
pdb|6PXV|A              QILLMFKTRPEDFRDLSFPKLIMITDYLLLFRVYGLESLKDLFPNLTVIRGSRLFFNYAL    93
                        ************************************************************

sp|P15208|INSR_MOUSE    VIFEMVHLKELGLYNLMNITRGSVRIEKNNELCYLATIDWSRILDSVEDNYIVLNKDDNE    180
pdb|6PXV|A              VIFEMVHLKELGLYNLMNITRGSVRIEKNNELCYLATIDWSRILDSVEDNYIVLNKDDNE    153
                        ************************************************************

sp|P15208|INSR_MOUSE    ECGDVCPGTAKGKTNCPATVINGQFVERCWTHSHCQKVCPTICKSHGCTAEGLCCHKECL    240
pdb|6PXV|A              ECGDICPGTAKGKTNCPATVINGQFVERCWTHSHCQKVCPTICKSHGCTAEGLCCHSECL    213
                        ****:***************************************************.***

sp|P15208|INSR_MOUSE    GNCSEPDDPTKCVACRNFYLDGQCVETCPPPYYHFQDWRCVNFSFCQDLHFKCRNSRKPG    300
pdb|6PXV|A              GNCSQPDDPTKCVACRNFYLDGRCVETCPPPYYHFQDWRCVNFSFCQDLHHKCKNSRRQG    273
                        ****:*****************:***************************.**:***: *

sp|P15208|INSR_MOUSE    CHQYVIHNNKCIPECPSGYTMNSSNLMCTPCLGPCPKVCQILEGEKTIDSVTSAQELRGC    360
pdb|6PXV|A              CHQYVIHNNKCIPECPSGYTMNSSNLLCTPCLGPCPKVCHLLEGEKTIDSVTSAQELRGC    333
                        **************************:************::*******************

sp|P15208|INSR_MOUSE    TVINGSLIINIRGGNNLAAELEANLGLIEEISGFLKIRRSYALVSLSFFRKLHLIRGETL    420
pdb|6PXV|A              TVINGSLIINIRGGNNLAAELEANLGLIEEISGYLKIRRSYALVSLSFFRKLRLIRGETL    393
                        *********************************:******************:*******

sp|P15208|INSR_MOUSE    EIGNYSFYALDNQNLRQLWDWSKHNLTITQGKLFFHYNPKLCLSEIHKMEEVSGTKGRQE    480
pdb|6PXV|A              EIGNYSFYALDNQNLRQLWDWSKHNLTITQGKLFFHYNPKLCLSEIHKMEEVSGTKGRQE    453

sp|P15208|INSR_MOUSE    RNDIALKTNGDQASCENELLKFSFIRTSFDKILLRWEPYWPPDFRDLLGFMLFYKEAPYQ    540
pdb|6PXV|A              RNDIALKTNGDQASCENELLKFSYIRTSFDKILLRWEPYWPPDFRDLLGFMLFYKEAPYQ    513
                        ***********************:************************************

sp|P15208|INSR_MOUSE    NVTEFDGQDACGSNSWTVVDIDPPQRSNDPKSQTPSHPGWLMRGLKPWTQYAIFVKTLVT    600
pdb|6PXV|A              NVTEFDGQDACGSNSWTVVDIDPPLRSNDPKSQ--NHPGWLMRGLKPWTQYAIFVKTLVT    571
                        ************************ ********  .************************

sp|P15208|INSR_MOUSE    FSDERRTYGAKSDIIYVQTDATNPSVPLDPISVSNSSSQIILKWKPPSDPNGNITHYLVY    660
pdb|6PXV|A              FSDERRTYGAKSDIIYVQTDATNPSVPLDPISVSNSSSQIILKWKPPSDPNGNITHYLVF    631
                        ***********************************************************:

sp|P15208|INSR_MOUSE    WERQAEDSELFELDYCLKGLKLPSRTWSPPFESDDSQKHNQSEYDDSASECCSCPKTDSQ    720
pdb|6PXV|A              WERQAEDSELFELDYCLKGLKLPSRTWSPPFESEDSQKHNQSEYEDSAGECCSCPKTDSQ    691
                        *********************************:**********:***.***********

sp|P15208|INSR_MOUSE    ILKELEESSFRKTFEDYLHNVVFVPRPSRKRRSLEEVGNVTATTLTLPDFPNVSSTIVPT    780
pdb|6PXV|A              ILKELEESSFRKTFEDYLHNVVFVPRPSRKRRSLGDVGNVTVAVPTVAAFPNTSSTSVPT    751
                        ********************************** :*****.:. *:  ***.*** ***

sp|P15208|INSR_MOUSE    SQEEHRPFEKVVNKESLVISGLRHFTGYRIELQACNQDSPDERCSVAAYVSARTMPEAKA    840
pdb|6PXV|A              SPEEHRPFEKVVNKESLVISGLRHFTGYRIELQACNQDTPEERCSVAAYVSARTMPEAKA    811
                        * ************************************:*:*******************

sp|P15208|INSR_MOUSE    DDIVGPVTHEIFENNVVHLMWQEPKEPNGLIVLYEVSYRRYGDEELHLCVSRKHFALERG    900
pdb|6PXV|A              DDIVGPVTHEIFENNVVHLMWQEPKEPNGLIVLYEVSYRRYGDEELHLCVSRKHFALERG    871
                        ************************************************************

sp|P15208|INSR_MOUSE    CRLRGLSPGNYSVRVRATSLAGNGSWTEPTYFYVTDYLDVPSNIAKIIIGPLIFVFLFSV    960
pdb|6PXV|A              CRLRGLSPGNYSVRIRATSLAGNGSWTEPTYFYVTDYLDVPSNIAKIIIGPLIFVFLFSV    931
                        **************:*********************************************
```

Supplementary Figure 2: INSR amino acid sequence alignment and 6PXV crystalline structure. * Shows fully protected root positions (:) It represents protection between groups with quite similar characteristics, (.) indicating protection between groups with low similarity characteristics.

EXCLI Journal 2022;21:400-414 – ISSN 1611-2156
Received: November 20, 2021, accepted: February 09, 2022, published: February 16, 2022

```
sp|P15208|INSR_MOUSE      VIGSIYLFLRKRQPDGPMGPLYASSNPEYLSASDVFPSSVYVPDEWEVPREKITLLRELG      1020
pdb|6PXV|A                VIGSIYLFLRKRQPDGPLGPLYASSNPEFLTASDVFPCSVYVPDEWEVSREKITLLRELG      991
                          *****************:**********:*:******.********** ***********

sp|P15208|INSR_MOUSE      QGSFGMVYEGNAKDIIKGEAETRVAVKTVNESASLRERIEFLNEASVMKGFTCHHVVRLL      1080
pdb|6PXV|A                QGSFGMVYEGNARDIIKGEAETRVAVKTVNESASLRERIEFLNEASVMKGFTCHHVVRLL      1051
                          ************:***********************************************

sp|P15208|INSR_MOUSE      GVVSKGQPTLVVMELMAHGDLKSHLRSLRPDAENNPGRPPPTLQEMIQMTAEIADGMAYL      1140
pdb|6PXV|A                GVVSKGQPTLVVMELMAHGDLKSYLRSLRPEAENNPGRPPPTLQEMIQMAAEIADGMAYL      1111
                          ***********************:******:******************:**********

sp|P15208|INSR_MOUSE      NAKKFVHRDLAARNCMVAHDFTVKIGDFGMTRDIYETDYYRKGGKGLLPVRWMSPESLKD      1200
pdb|6PXV|A                NAKKFVHRNLAARNCMVAHDFTVKIGDFGMTRDIYETDYYRKGGKGLLPVRWMAPESLKD      1171
                          ********:********************************************:******

sp|P15208|INSR_MOUSE      GVFTASSDMWSFGVVLWEITSLAEQPYQGLSNEQVLKFVMDGGYLDPPDNCPERLTDLMR      1260
pdb|6PXV|A                GVFTTSSDMWSFGVVLWEITSLAEQPYQGLSNEQVLKFVMDGGYLDQPDNCPERVTDLMR      1231
                          ****:***************************************** *******:*****

sp|P15208|INSR_MOUSE      MCWQFNPKMRPTFLEIVNLLKDDLHPSFPEVSFFYSEENKAPESEELEMEFEDMENVPLD      1320
pdb|6PXV|A                MCWQFNPKMRPTFLEIVNLLKDDLHPSFPEVSFFHSEENKAPESEELEMEFEDMENVPLD      1291
                          **********************************:*************************

sp|P15208|INSR_MOUSE      RSSHCQREEAGGREGGSSLSIKRTYDEHIPYTHMNGGKKNGRVLTLPRSNPS--------      1372
pdb|6PXV|A                RSSHCQREEAGGRDGGSSLGFKRSYEEHIPYTHMNGGKKNGAAATAPRSNPSLESSGLEV      1351
                          *************:*****.:**:*:*************** . * ******

sp|P15208|INSR_MOUSE      ---     1372
pdb|6PXV|A                LFQ     1354
```

Supplementary Figure 2 (cont.): INSR amino acid sequence alignment and 6PXV crystalline structure. * Shows fully protected root positions (:) It represents protection between groups with quite similar characteristics, (.) indicating protection between groups with low similarity characteristics.

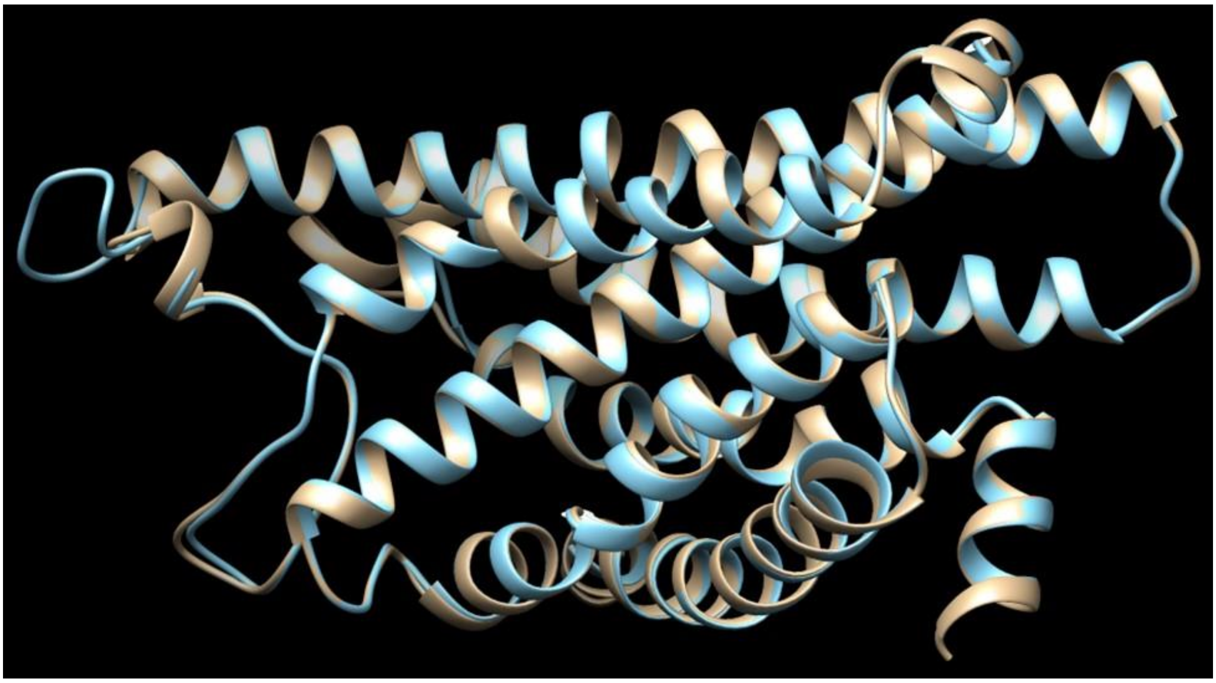

Supplementary Figure 3: Alignment of CMKLR1 model (cyan color) and 6OMM template structure of N-formyl peptide receptor 2 using Chimera software, this model showed that template structure and model have high structural similarity with low RMSD value.

EXCLI Journal 2022;21:400-414 – ISSN 1611-2156
Received: November 20, 2021, accepted: February 09, 2022, published: February 16, 2022

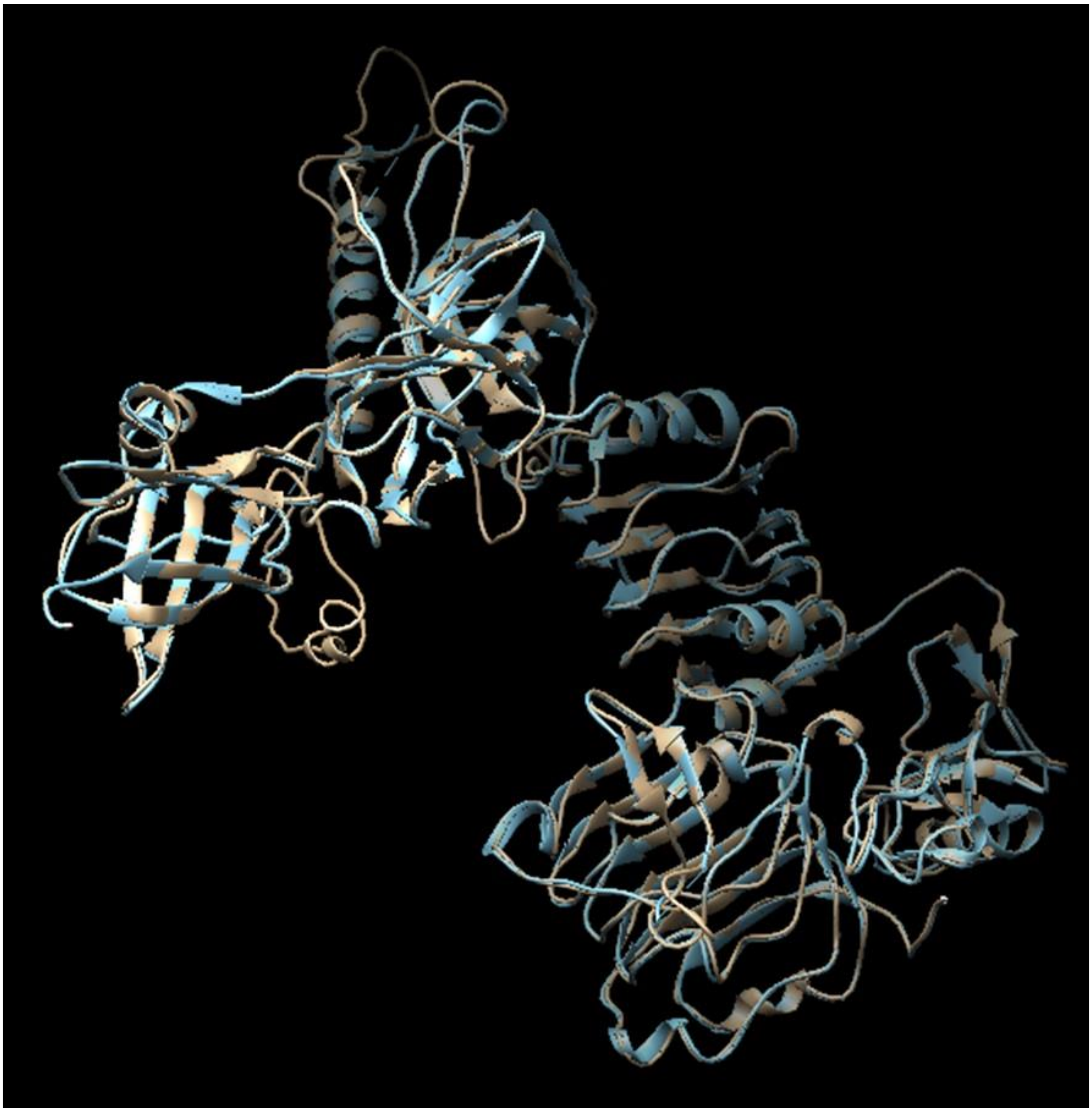

Supplementary Figure 4: INSR model Alignment and 6PXV template structure of insulin receptor protein using Chimera, this model showed that the template structure and model have high structural similarity with low RMSD values.

EXCLI Journal 2022;21:400-414 – ISSN 1611-2156
Received: November 20, 2021, accepted: February 09, 2022, published: February 16, 2022

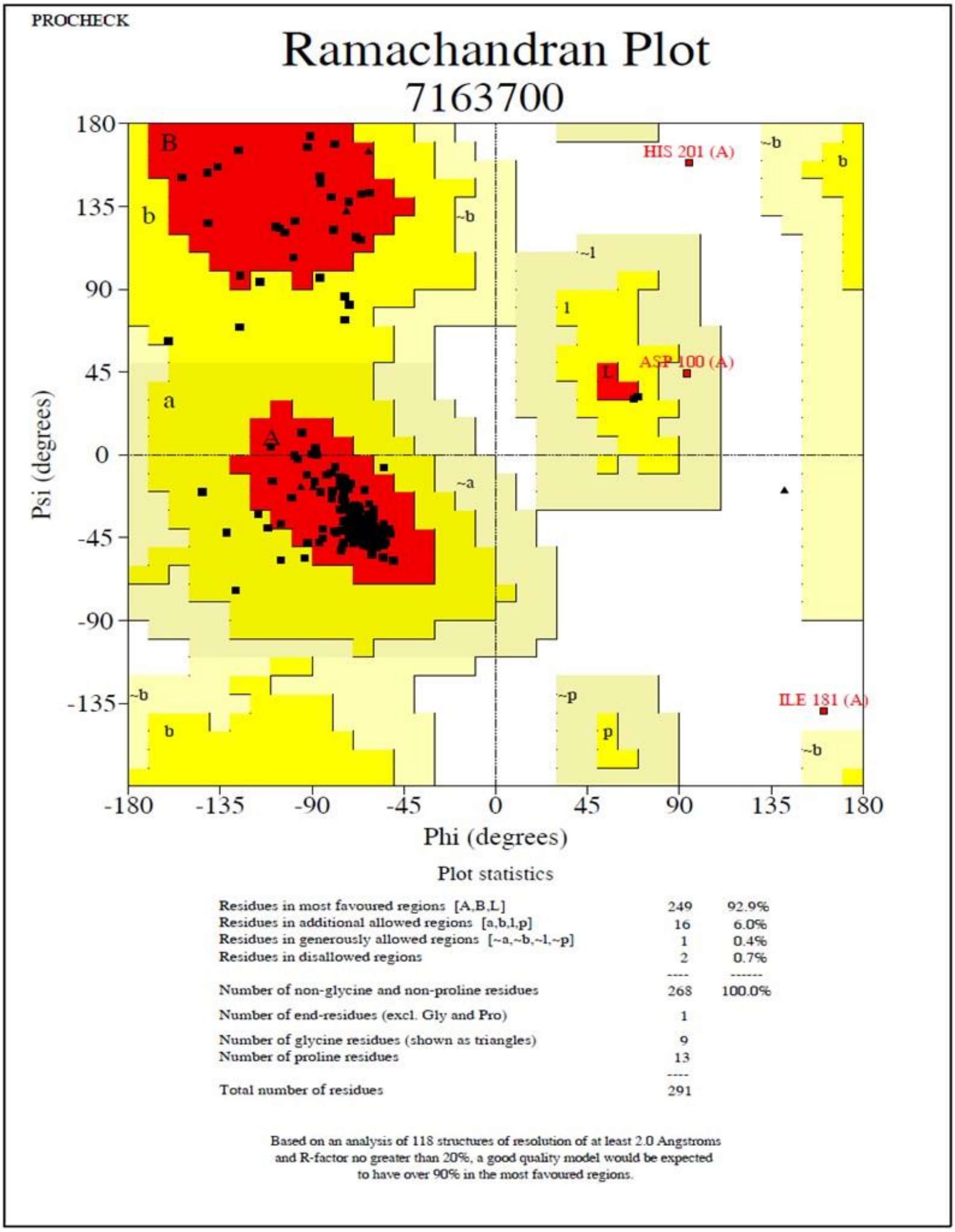

Supplementary Figure 5: Evaluation of stereochemical accuracy of CMKLR1 model by Ramachandran plot. This plot shows that 92.9 % (249 amino acids) of residues in favored regions, 6.0 % (16 amino acids) in additional allowed regions, 0.4 % (1 amino acid) in generously allowed regions and 0.7 % (2 amino acids) in disallowed regions and Ramachandran plot shows most of the amino acids of the predicted model are in the favored and allowed regions and indicate the good stereochemical quality of the model.

EXCLI Journal 2022;21:400-414 – ISSN 1611-2156
Received: November 20, 2021, accepted: February 09, 2022, published: February 16, 2022

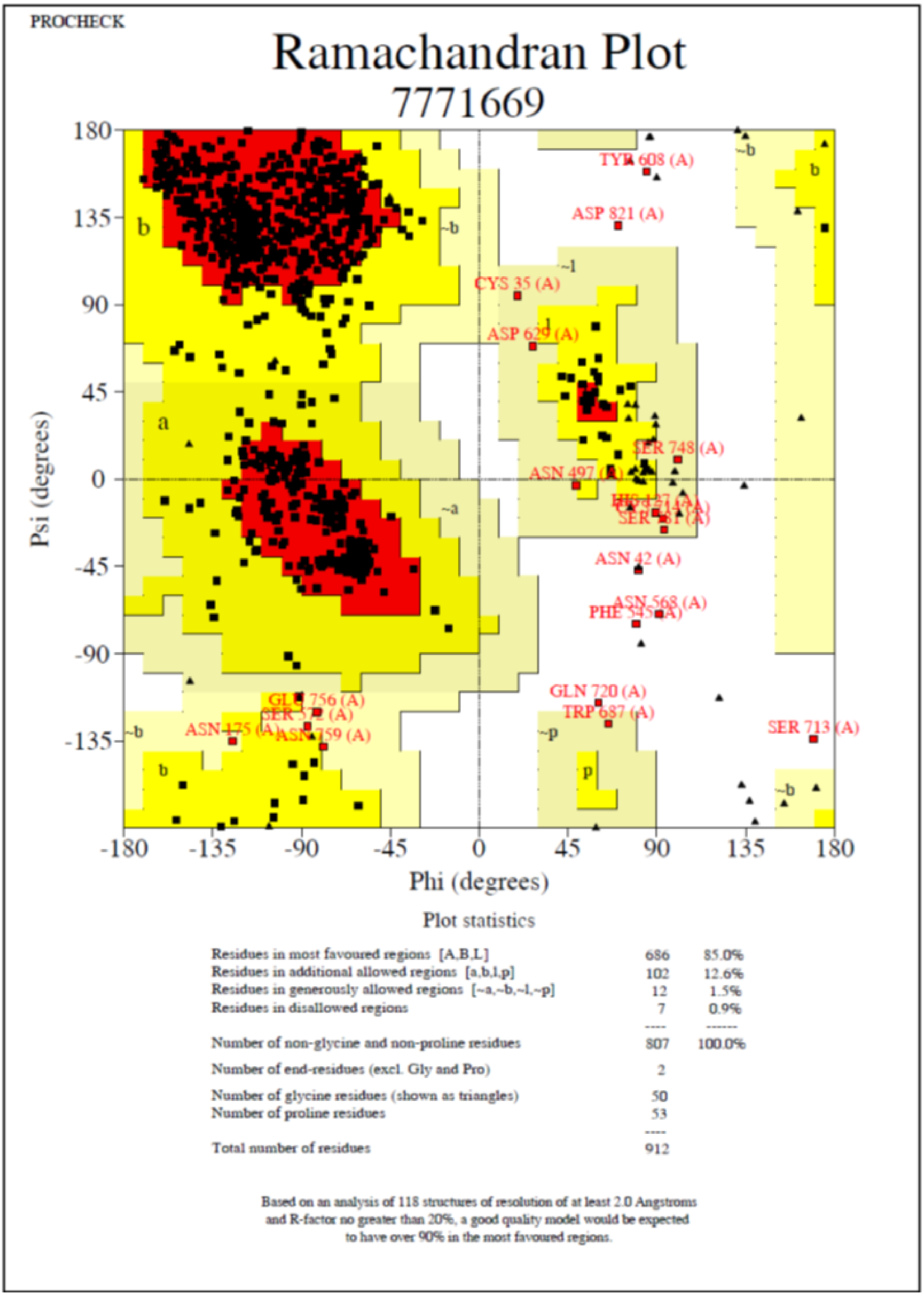

Supplementary Figure 6: Evaluation of stereochemical accuracy of INSR model by Ramachandran plot. This plot shows that 85 % (686 amino acids) of residues in favored regions, 12.6 % (102 amino acids) in additional allowed regions, 1.5 % (12 amino acid) in generously allowed regions and 0.9 % (7 amino acids) in disallowed regions and Ramachandran plot shows most of the amino acids of the predicted model are in the favored and allowed regions and indicate the good stereochemical quality of the model.

EXCLI Journal 2022;21:400-414 – ISSN 1611-2156
Received: November 20, 2021, accepted: February 09, 2022, published: February 16, 2022

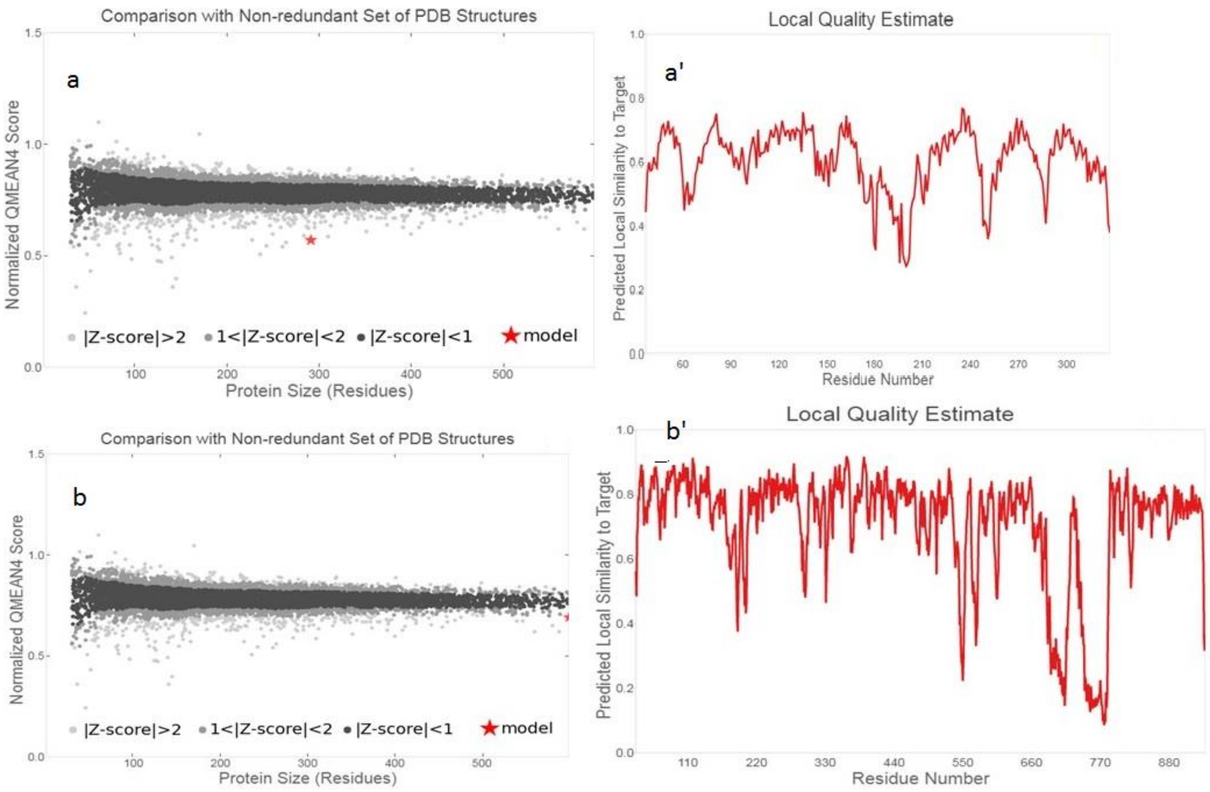

Supplementary Figure 7: Structure validation of modeled CMKLR1 and INSR protein structures: (**a** and **b**) Comparison of the modeled protein structures with a non-redundant set of PDB structures. (**a´** and **b´**). Local quality estimate of the residue graphs.

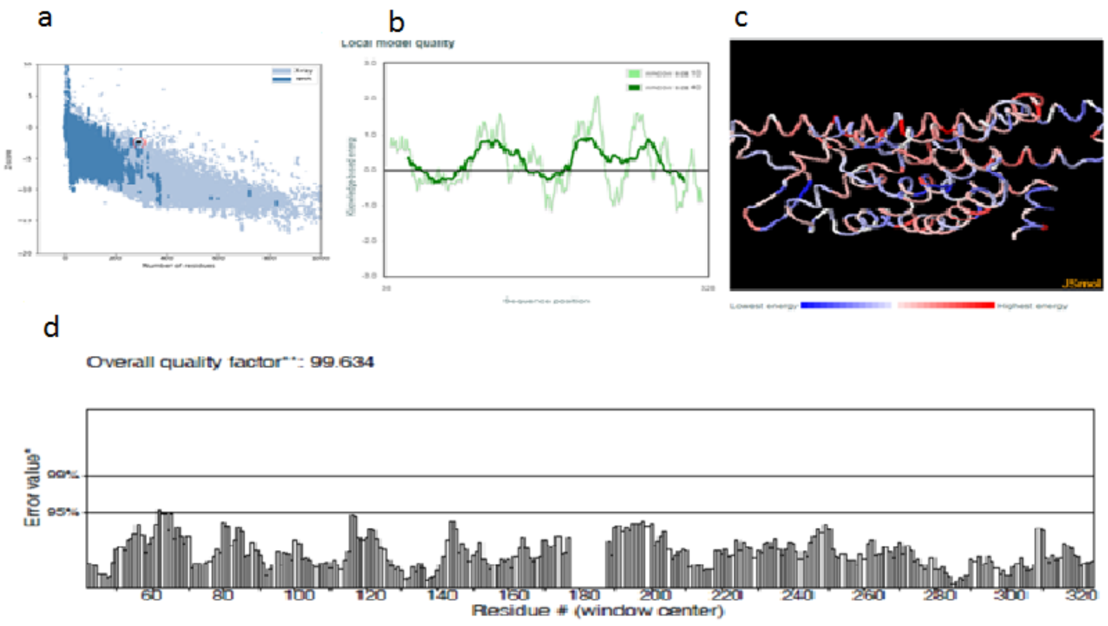

Supplementary Figure 8: (**a,b,c**) Validation of CMKLR1 model. Z-score diagram is surface energy diagram of amino acids and surface energy display on the 3D structure of the protein, (**d**) ERRAT energy plan

EXCLI Journal 2022;21:400-414 – ISSN 1611-2156
Received: November 20, 2021, accepted: February 09, 2022, published: February 16, 2022

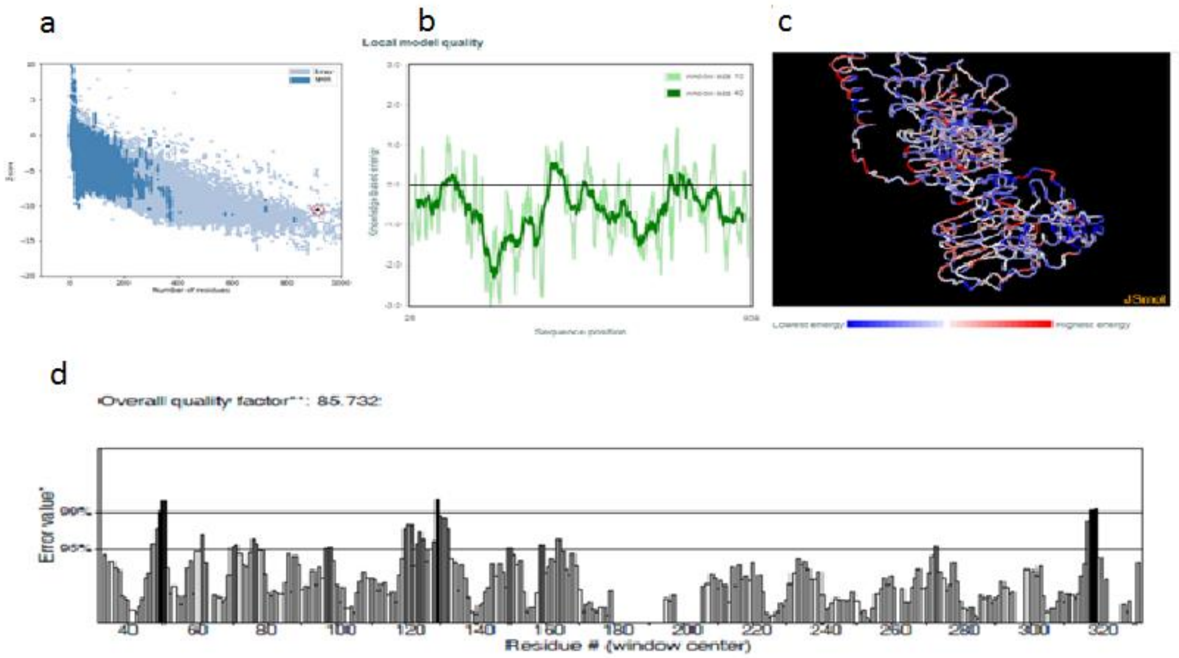

Supplementary Figure 9: (a,b,c) Validation of the INSR model. The Z-score diagram is a surface energy diagram of amino acids and surface energy display on the 3D structure of the protein, (**d**) ERRAT energy plan

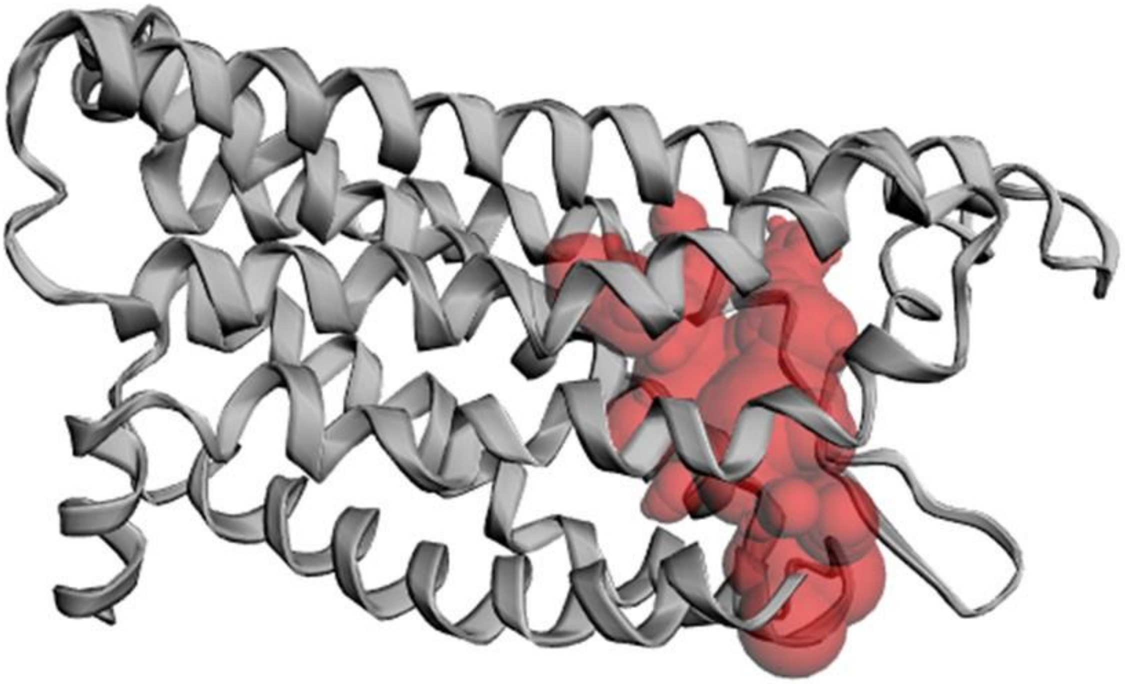

Supplementary Figure 10: Binding envelope surface of CMKLR1 modeled protein calculated using CASTp 3.0. Docking analysis confirms the binding envelope level of proteins calculated using CASTp in CMKLR1 protein.

EXCLI Journal 2022;21:400-414 – ISSN 1611-2156
Received: November 20, 2021, accepted: February 09, 2022, published: February 16, 2022

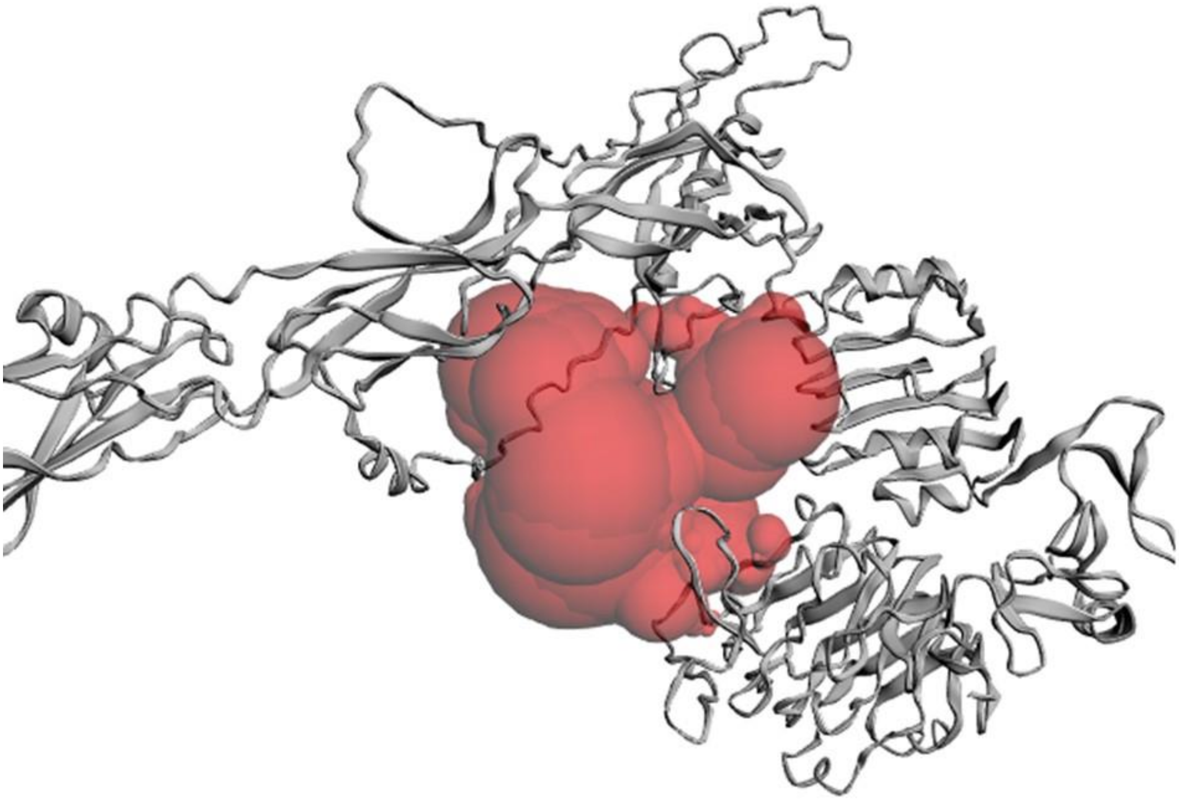

Supplementary Figure 11: Level binding envelope of modeled proteins calculated using CASTp 3.0. Docking analysis confirms the binding envelope level of proteins calculated using CASTp in INSR proteins.

EXCLI Journal 2022;21:400-414 – ISSN 1611-2156
Received: November 20, 2021, accepted: February 09, 2022, published: February 16, 2022

Supplementary Table 1: E-value and sequence identity of CMKLR1 and INSR in the different programs

server	E-value	sequence identity (%)
**PDB-Blast**	6e-61 (CMKLR1)<br>0.0 (INSR)	35.19 (CMKLR1)<br>95.64 (INSR)
**JPRED**	7e-52 (CMKLR1)<br>0.0 (INSR)	-
**Phyre2**	-	32.14 (CMKLR1)<br>92.17 (INSR)

Supplementary Table 2: Serum hormones profile of studied groups

Group	FSH (IU/L)	LH (IU/L)	T (ng/mL)	E2 (pg/ml)
**Control**	0.08±0.01	0.08±0.01	0.011±0.002	18±0.8
**PCOS model**	0.06±0.02	0.09±0.02	0.015±0.002	30±2*
**Control minocycline**	0.07±0.01	0.06±0.01	0.013±0.002	14±2.5
**PCOS minocycline**	0.05±0.01	0.09±0.03	0.014±0.003	16±0.5**
**PCOS letrozole**	0.15±0.04	0.18±0.04	0.006±0.002	14±2†
**PCOS metformin**	0.13±0.03	0.15±0.03	0.024±0.002	16±0.12†

NOTE: * compared to control group; ** compared to control minocycline group; † compared to PCOS model group. Estradiol concentration significantly increased in PCOS model group compared to the control group (*P<0.01) Also, (**P<0.01) PCOS Minocycline vs. PCOS model. (†P<0.01) PCOS Letrozole vs. PCOS model. (†P<0.01) PCOS Metformin vs. PCOS model.

Supplementary Table 3: Evaluation of different ovarian follicle stages in studied groups

Group	Primary F	Secondary F	Graafian F	Corpus Luteum	Hemorrhagic F
**Control**	3±1	7±1	3±1	5±2	2±1
**PCOS model**	4±1	10±4	8±2*	1±1*	3±1
**Control minocycline**	2±1	4±1	3±1	3±2	2±1
**PCOS minocycline**	3±1	6±1	2±1†	4±1†	2±1
**PCOS letrozole**	2±1	4±2	2±1†	1±1	6±2†
**PCOS metformin**	2±1	4±1	2±1†	3±1†	2±1

NOTE: * compared to control group; ** compared to control minocycline group; † compared to PCOS model group. The number of Graafian follicles were significantly decreased in the PCOS Minocycline, PCOS Letrozole, PCOS Metformin vs. PCOS model (†P < 0.01), and increased PCOS model vs. control(*P<0.01) . The number of Corpus Luteum were significantly increased in the PCOS Minocycline vs. PCOS model (†P < 0.01), and PCOS metformin vs PCOS model. (†P <0.01), and decreased PCOS model vs. control(*P<0.001).
