# Supplementary material for: Molecular docking and mouse modeling suggest CMKLR1 and INSR as targets for improving PCOS phenotypes by minocycline
Source: EXCLI J. 2022 Feb 16;21:400–14. doi: 10.17179/excli2021-4534 (PMC8971357; doi:10.17179/excli2021-4534)
Supplement: Supplementary data [file EXCLI-21-400-s-002.pdf]

EXCLI Journal 2022;21:400-414 – ISSN 1611-2156
Received: November 20, 2021, accepted: February 09, 2022, published: February 16, 2022

Supplementary data to:

Original article:

MOLECULAR DOCKING AND MOUSE MODELING SUGGEST CMKLR1 AND INSR AS TARGETS FOR IMPROVING PCOS PHENOTYPES BY MINOCYCLINE

Mahdie Kian[a†], Elham Hosseini[b†], Tooba Abdizadeh[c], Taimour Langaee[d], Azadeh Khajouei[a], Sorayya Ghasemi[a*]

[a] Cellular and Molecular Research Center, Basic Health Sciences Institute, Shahrekord University of Medical Sciences, Shahrekord, Iran
[b] Department of Obstetrics and Gynecology, IVF Clinic, Mousavi Hospital, School of Medicine, Zanjan University of Medical Sciences, Zanjan, Iran
[c] Clinical Biochemistry Research Center, Basic Health Sciences Institute, Shahrekord University of Medical Sciences, Shahrekord, Iran
[d] Center for Pharmacogenomics and Precision Medicine, College of Pharmacy, University of Florida, Gainesville, FL, USA

[†] Joint first authors, contributed equally to this research work.

* **Corresponding author:** Sorayya Ghasemi, Ph.D., Associate Professor, Cellular and Molecular Research Center, Basic Health Sciences Institute, Shahrekord University of Medical Sciences, Shahrekord, Iran; Tel: 09131856090, 03833331471;
E-mails: s.ghasemi@skums.ac.ir, sorayya.ghasemi@gmail.com

https://dx.doi.org/10.17179/excli2021-4534

This is an Open Access article distributed under the terms of the Creative Commons Attribution License (http://creativecommons.org/licenses/by/4.0/).

EXCLI Journal 2022;21:400-414 – ISSN 1611-2156
Received: November 20, 2021, accepted: February 09, 2022, published: February 16, 2022

Raw data of Supplementary Table 2:

The levels of PCOS-related hormones were measured in all mice in different groups with ELISA immediately before killing the mice. The levels of Follicle Stimulating Hormone (FSH), Luteinizing Hormone (LH), Estradiol (E2) and Testosterone (T), were compared with the metformin group to the other studied groups.

Groups: 1: Control; 2: PCOS model; 3: Minocycline control; 4: PCOS minocycline; 5: PCOS letrozole; 6: PCOS metformin

Groups	E2	FSH	LH	T
**1**	19.80	0.09	0.09	0.011
**1**	13.00	0.04	0.07	0.013
**1**	18.20	0.15	0.02	0.009
**1**	17.50	0.09	0.07	0.009
**1**	19.90	0.08	0.15	0.010
**1**	18.20	0.08	0.08	0.009
**1**	17.50	0.09	0.09	0.007
**1**	19.60	0.07	0.07	0.027
**2**	37.00	0.03	0.03	0.009
**2**	27.00	0.19	0.04	0.015
**2**	24.00	0.05	0.15	0.008
**2**	37.00	0.02	0.09	0.010
**2**	34.00	0.07	0.11	0.017
**2**	35.00	0.02	0.19	0.020
**2**	28.10	0.08	0.06	0.022
**2**	23.00	0.04	0.06	0.020
**3**	6.00	0.13	0.07	0.019
**3**	20.30	0.03	0.13	0.019
**3**	21.20	0.05	0.05	0.016
**3**	5.80	0.07	0.07	0.006
**3**	16.40	0.07	0.05	0.009
**3**	6.80	0.06	0.07	0.015
**3**	23.10	0.08	0.03	0.015
**3**	13.10	0.08	0.06	0.006
**4**	16.50	0.04	0.28	0.019
**4**	12.50	0.06	0.05	0.017
**4**	16.70	0.04	0.04	0.029
**4**	16.30	0.04	0.17	0.017
**4**	15.90	0.02	0.04	0.005
**4**	16.90	0.07	0.04	0.011
**4**	15.90	0.13	0.06	0.003
**4**	17.40	0.06	0.05	0.016
**5**	9.50	0.15	0.05	0.020
**5**	19.40	0.11	0.21	0.004
**5**	10.00	0.09	0.18	0.003
**5**	22.70	0.04	0.39	0.003
**5**	10.00	0.11	0.15	0.008

EXCLI Journal 2022;21:400-414 – ISSN 1611-2156
Received: November 20, 2021, accepted: February 09, 2022, published: February 16, 2022

**5**	10.30	0.30	0.14	0.007
**5**	10.20	0.34	0.30	0.003
**5**	20.10	0.06	0.09	0.007
**6**	16.01	0.16	0.11	0.025
**6**	15.90	0.04	0.07	0.010
**6**	16.22	0.05	0.10	0.029
**6**	15.50	0.09	0.29	0.024
**6**	16.03	0.07	0.10	0.023
**6**	15.90	0.22	0.19	0.025
**6**	16.60	0.29	0.27	0.030
**6**	15.83	0.12	0.11	0.027

EXCLI Journal 2022;21:400-414 – ISSN 1611-2156
Received: November 20, 2021, accepted: February 09, 2022, published: February 16, 2022

Raw data of Supplementary Table 3:

The different types of follicles were counted. The counting of different types of follicles (primary, secondary, antral, and atretic follicles) and corpus luteum (CL) in the studied groups were shown in this raw data table. Some slide images of each group are shown randomly in the article. Each number in this table is the average of the follicles' number of three randomly selected slides prepared from the ovary of each mouse.

Groups: 1: Control; 2: PCOS model; 3: Minocycline control; 4: PCOS minocycline; 5: PCOS letrozole; 6: PCOS metformin

Groups	Primary Follicles	Secondary Follicles	Graafian Follicles	Corpus Luteum	Hemorrhagic Follicles
**1**	3.00	6.00	2.00	4.00	4.00
**1**	2.00	7.00	3.00	6.00	2.00
**1**	2.00	8.00	3.00	9.00	3.00
**1**	3.00	8.00	2.00	4.00	1.00
**1**	4.00	6.00	3.00	5.00	2.00
**1**	3.00	7.00	5.00	2.00	1.00
**1**	5.00	6.00	4.00	6.00	2.00
**1**	2.00	9.00	2.00	4.00	2.00
**2**	4.00	12.00	9.00	0.00	3.00
**2**	5.00	12.00	7.00	1.00	5.00
**2**	4.00	5.00	8.00	3.00	3.00
**2**	2.00	13.00	8.00	0.00	2.00
**2**	5.00	14.00	8.00	2.00	2.00
**2**	4.00	13.00	4.00	0.00	3.00
**2**	3.00	4.00	11.00	1.00	2.00
**2**	5.00	7.00	9.00	1.00	4.00
**3**	3.00	4.00	5.00	0.00	3.00
**3**	2.00	3.00	3.00	3.00	3.00
**3**	3.00	5.00	4.00	3.00	2.00
**3**	3.00	4.00	2.00	2.00	3.00
**3**	2.00	5.00	2.00	3.00	2.00
**3**	1.00	4.00	3.00	7.00	3.00
**3**	2.00	5.00	2.00	2.00	0.00
**3**	0.00	2.00	3.00	4.00	1.00
**4**	4.00	5.00	2.00	5.00	3.00
**4**	5.00	6.00	3.00	4.00	1.00
**4**	3.00	7.00	3.00	4.00	3.00
**4**	3.00	6.00	2.00	3.00	1.00
**4**	2.00	6.00	0.00	5.00	3.00
**4**	2.00	7.00	3.00	4.00	3.00
**4**	2.00	7.00	1.00	2.00	1.00
**4**	3.00	4.00	2.00	5.00	1.00
**5**	2.00	0.00	2.00	0.00	9.00
**5**	3.00	4.00	2.00	1.00	4.00
**5**	2.00	6.00	3.00	1.00	5.00

EXCLI Journal 2022;21:400-414 – ISSN 1611-2156
Received: November 20, 2021, accepted: February 09, 2022, published: February 16, 2022

**5**	3.00	5.00	0.00	1.00	8.00
**5**	0.00	6.00	3.00	0.00	6.00
**5**	2.00	4.00	1.00	2.00	4.00
**5**	1.00	5.00	2.00	0.00	4.00
**5**	3.00	2.00	3.00	3.00	8.00
**6**	2.00	3.00	0.00	2.00	1.00
**6**	0.00	4.00	2.00	2.00	1.00
**6**	2.00	4.00	3.00	5.00	4.00
**6**	3.00	5.00	1.00	2.00	1.00
**6**	3.00	2.00	2.00	3.00	3.00
**6**	2.00	4.00	3.00	4.00	2.00
**6**	3.00	5.00	3.00	3.00	2.00
**6**	1.00	5.00	2.00	3.00	2.00

EXCLI Journal 2022;21:400-414 – ISSN 1611-2156
Received: November 20, 2021, accepted: February 09, 2022, published: February 16, 2022

Raw data of Figure 4:

Expression level of CMKLR1 and INSR genes compared to control gene for each mouse in each group are shown in this raw data table.

Groups: 1: Control; 2: PCOS model; 3: Minocycline control; 4: PCOS minocycline; 5: PCOS letrozole; 6: PCOS metformin. Group 5 is non-functional as a positive control for INSR expression level. Hence, this group only was measured for the amount of CMKLR1expression level.

Groups	CMKLR1 expression level	INSR expression level
**1**	1.18	1.00
**1**	1.02	0.67
**1**	0.99	0.80
**1**	0.84	0.75
**1**	0.87	0.97
**1**	1.13	1.00
**1**	1.08	0.86
**1**	0.91	0.78
**2**	3.03	1.90
**2**	2.93	1.57
**2**	3.69	1.96
**2**	3.56	2.42
**2**	2.65	2.45
**2**	3.40	1.40
**2**	3.80	1.30
**2**	2.83	1.02
**3**	1.27	1.67
**3**	0.44	2.37
**3**	0.75	2.82
**3**	1.40	1.20
**3**	1.44	1.10
**3**	0.32	3.02
**3**	0.30	3.15
**3**	0.60	0.90
**4**	1.08	3.04
**4**	1.00	5.40
**4**	1.04	7.13
**4**	1.07	8.30
**4**	1.07	6.30
**4**	1.02	2.13
**4**	1.06	2.40
**4**	1.04	1.20
**5**	0.10	-
**5**	0.22	-
**5**	0.12	-
**5**	0.25	-
**5**	0.10	-

EXCLI Journal 2022;21:400-414 – ISSN 1611-2156
Received: November 20, 2021, accepted: February 09, 2022, published: February 16, 2022

**5**	0.24	-
**5**	0.30	-
**5**	0.09	-
**6**	0.06	0.84
**6**	0.05	0.89
**6**	0.08	0.85
**6**	0.07	0.79
**6**	0.07	0.89
**6**	0.06	0.83
**6**	0.07	0.86
**6**	0.06	0.84

EXCLI Journal 2022;21:400-414 – ISSN 1611-2156
Received: November 20, 2021, accepted: February 09, 2022, published: February 16, 2022

Raw data of mice weight:

Weight of mice in each group; the body weight of mice in all groups was measured weekly. In this raw data table, the body weights of mice prior to the start of study, after PCOS induction, and at the end of treatment were shown.

Groups: 1: Control; 2: PCOS model; 3: Minocycline control; 4: PCOS minocycline; 5: PCOS letrozole; 6: PCOS metformin

Groups	Weight at the beginning of the study	Weight after PCOS induction (gr)	Weight After treatment (gr)
1	15.30	17.30	21.00
1	15.10	16.40	19.60
1	15.40	17.20	20.10
1	14.90	19.00	23.00
1	14.50	21.00	23.90
1	14.90	20.00	25.10
1	12.30	15.10	19.60
1	14.20	17.00	21.10
2	14.70	18.00	20.30
2	13.80	19.00	23.60
2	12.60	15.00	19.50
2	15.70	21.00	24.80
2	15.90	18.70	22.60
2	12.40	15.00	22.10
2	15.70	17.00	23.00
2	14.30	16.20	21.20
3	13.90	17.00	21.20
3	14.60	16.30	21.70
3	14.70	17.00	23.00
3	14.90	21.10	23.20
3	15.20	18.00	22.40
3	13.90	19.00	22.70
3	15.90	18.00	23.40
3	14.70	16.00	21.20
4	15.70	15.00	22.60
4	15.30	19.00	23.80
4	13.90	16.30	23.50
4	14.60	20.20	23.30
4	14.50	18.00	24.20
4	15.00	17.00	23.60
4	16.00	20.00	24.70
4	6.10	19.00	23.60
5	14.90	17.20	23.80
5	14.50	17.00	23.00
5	12.90	15.00	24.40

EXCLI Journal 2022;21:400-414 – ISSN 1611-2156
Received: November 20, 2021, accepted: February 09, 2022, published: February 16, 2022

**5**	15.90	19.50	24.00
**5**	15.00	18.00	24.50
**5**	16.20	20.00	25.50
**5**	16.80	20.30	23.00
**5**	13.80	17.00	22.70
**6**	13.10	15.00	22.50
**6**	14.40	18.00	23.10
**6**	16.60	21.00	26.80
**6**	12.80	15.80	19.70
**6**	14.30	17.00	23.10
**6**	16.30	20.40	26.10
**6**	13.10	16.00	24.10
**6**	13.40	16.40	24.70
